# Multimode sensing based on optical microcavities

**DOI:** 10.1007/s12200-023-00084-1

**Published:** 2023-10-27

**Authors:** Yanran Wu, Bing Duan, Changhong Li, Daquan Yang

**Affiliations:** 1 State Key Laboratory of Information Photonics and Optical Communications, Beijing University of Posts and Telecommunications, Beijing, 100876 China; 2 School of Information and Communication Engineering, Beijing University of Posts and Telecommunications, Beijing, 100876 China; 3 School of Electronic Information, Qingdao University, Qingdao, 266071 China

**Keywords:** Optical microcavity, Multimode sensing, Multiparameter measurement, Sensing mechanisms

## Abstract

Optical microcavities have the ability to confine photons in small mode volumes for long periods of time, greatly enhancing light-matter interactions, and have become one of the research hotspots in international academia. In recent years, sensing applications in complex environments have inspired the development of multimode optical microcavity sensors. These multimode sensors can be used not only for multi-parameter detection but also to improve measurement precision. In this review, we introduce multimode sensing methods based on optical microcavities and present an overview of the multimode single/multi-parameter optical microcavities sensors. Expected further research activities are also put forward.

**Graphical abstract:**

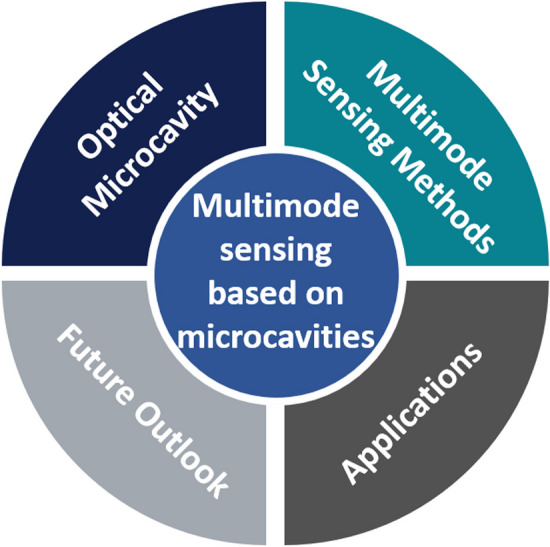

## Introduction

In recent years, optical sensing technologies have been widely applied in biomedical research, environmental monitoring, and national security due to their advantages of being label-free and resistant to electromagnetic interference [1–3]. Various optical sensors, such as surface plasmon resonance, optical waveguides, photonic crystals, and optical microcavities, have been proven effective. Optical microcavities with high quality (*Q*) factors and small mode volumes (*V*) can greatly enhance the light-matter interaction [4], leading to unprecedented levels of sensitivity and low detection limits. Therefore, optical microcavities have been widely employed in various sensing applications, including biosensing [5–10], chemical sensing [11–13], and sensing of various physical quantities [14–16].

The main types of optical microcavities, Fabry–Perot (FP) cavities, whispering gallery mode (WGM) cavities, and photonic crystal (PhC) cavities have been playing crucial roles in the field of sensing. For instance, FP microcavities have been used for biomolecular detection [6, 17, 18]. WGM microcavities, combined with localized surface plasmon resonance enhancement and other techniques, have achieved single-molecule or even single-ion detection levels [19–21]. PhC microcavities have been applied in capturing single nanoparticle and label-free molecule detection [22–24]. However, the aforementioned detections are limited to single-parameter measurements. In practical applications, the target parameters are often the result of multiple effects acting together. Conventional optical microcavity sensing approaches, such as monitoring the changes in individual resonant modes, have difficulties in fully utilizing spectral information. Additionally, such approaches are unable to achieve independent decoupling and real-time measurement of multiple parameters. Therefore, there is a pressing demand for reliable and versatile multimode sensing techniques.

Sensing applications in complex environments have driven research on optical microcavity sensors for multimode detection. Multimode sensing offers several advantages over single-mode sensing. First, multimode sensors can be used for multi-parameter sensing. The principle behind multimode sensing is that different resonant modes in the microcavity spectrum respond differently to different parameters, effectively acting as multiple sensors. Therefore, multimode sensing can fully utilize the sensing information from different modes, providing a solution for high-precision parallel detection of multiple parameters. For example, real-time decoupling and independent measurement of multiple parameters have been achieved through sensing methods based on multiparameter sensing matrices [25] and self-reference [26]. Additionally, multimode sensors can be used for single-parameter measurement by monitoring the collective behaviors of different modes, further improving the detection limits and enabling wide-range parameter measurements. High-precision and wide-range temperature measurements have been achieved using optical barcodes [27], while machine-learning techniques have been employed for accurate pressure measurements [28].

This paper provides a comprehensive review of the latest research advances in multimode optical microcavity sensing, covering both single-parameter sensing and multiparameter sensing. Section 2 provides discussion of commonly used multimode sensing methods that aim to improve detection limits and to enable wide-range and multiparameter sensing measurements. In Sect. 3, the applications of multimode sensing in single/multi-parameter sensing are outlined. Finally, in Sect. 4, the challenges and future development directions of multimode microcavity sensing are summarized.

## Sensing mechanisms of multimode sensors based on optical microcavities

**Fig. 1:**
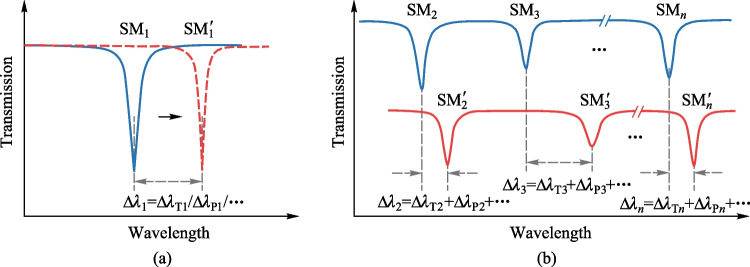
**a** Schematic diagram of single-mode single-parameter principle. The arrow indicates that the resonator mode has shifted. **b** Schematic diagram of multi-mode single/multi-parameter principle.

Conventional microcavity sensing schemes focus on individual parametric sensing of a single resonant mode. Mode shifts (∆*λ*_1_,…, ∆*λ*_*n*_) are used as an example to describe the principle of single-mode single-parameter sensing and multi-mode single/multi-parameter sensing. The principle of single-mode single-parameter sensing is shown in Fig. 1a. When the external temperature/pressure parameter changes, the sensing mode shifts, and the spectral position changes from SM_1_ to SM_1_′, resulting in a spectral offset ∆*λ*_1_, which is determined by the temperature/pressure change. That is, when the temperature is changed, ∆*λ*_1_ = ∆*λ*_T1_; When the pressure is changed, ∆*λ*_1_ = ∆*λ*_P1_. Thus, the relative temperature/pressure (∆*T*/∆*P*) parameter is sensed by detecting the relative displacement ∆*λ*_1_ of the resonant mode SM_1_. However, a single resonant mode can only sense one parameter at a time, limiting its application in complex environments.

The principle of multi-mode single/multi-parameter sensing is shown in Fig. 1b. Due to the different responses of multiple resonant modes (SM_2_,…, SM_*n*_) to the parameter, parametric information can be obtained from the collective behavior of multiple resonant modes. When multiple modes perceive a single parameter, that parameter uniquely determines the offset of the overall mode of the spectrum. For example, multiple sensing modes are shifted when the temperature is altered. The spectral position is changed from (SM_2_,…, SM_*n*_) to (SM_2_′,…, SM_*n*_′), and the corresponding spectral offsets (∆*λ*_2_,…, ∆*λ*_*n*_) are temperature-induced, i.e., ∆*λ*_2_ = ∆*λ*_T2_, ∆*λ*_3_ = ∆*λ*_T3_,…, ∆*λ*_*n*_ = ∆*λ*_T*n*_. Therefore, we can derive this parameter from the overall pattern of the spectrum. When multiple modes perceive multiple parameters at the same time, multiple parameters jointly determine the shift of the overall mode of the spectrum. For example, when temperature and pressure are changed, each sensing mode in (SM_2_,…, SM_*n*_) is affected by both temperature and pressure. The offsets of the sensing modes due to temperature and pressure are ∆*λ*_T_ and ∆*λ*_P_, respectively, and the final offset of the sensing mode is the sum of the two offsets, ∆*λ* = ∆*λ*_T_ + ∆*λ*_P_. Then it is necessary to combine some data processing methods to decouple multiple parameters independently. For the processing of complex multimode sensing signals, methods such as multiparameter sensing matrix [25] and machine learning [28] have been reported. The multiparameter sensing matrix utilizes the linear relationship between different parameters and mode offsets to achieve independent decoupling and parallel measurement of multiple parameters. Machine learning-based multimode optical microcavity sensors utilize advanced algorithms to analyze the multimode shift data of the sensors. This establishes the mapping relationship between the optical data and the optical response, to provide accurate and reliable predictions for single/multiparameter measurements. In addition, other features of multimode spectra, such as resonance wavelength, number of resonance modes, mode spacing, and mode linewidth, can also be used as effective sensing information to train sensing models. In this section, we briefly review the principles of these multimode-sensing mechanisms.

### Multiparameter sensing matrix

The multiparameter sensor matrix is a commonly used method for multiparameter sensing. Generally, each mode in the spectrum shows a different response to the target parameter. Therefore, the interaction between analytes and the sensor can be converted into changes in the resonant wavelength, enabling multiple parameter measurements. The multiparameter sensor matrix consists of a sensitivity matrix, a resonant wavelength shift matrix, and a relative change matrix of the target parameters to be measured, where the sensitivity matrix (*M*) must be invertible. The sensitivity matrix *M* consists of the sensitivity of different modes to different parameters (*S*_11_,…, *S*_*nn*_), and is defined as follows [29]:

1 $$M=\left[ \begin{array}{ccc}{S}_{11}& \cdots & {S}_{n1}\\ \cdots & \cdots & \cdots \\ {S}_{1n}& \cdots & {S}_{nn}\end{array}\right].$$

The wavelength shifts of the modes (∆*λ*_1_,…, ∆*λ*_*n*_) induced by different parameter variations (∆*v*_1_,…, ∆*v*_*n*_) are given by

2 $$\left[\begin{array}{c}\Delta {\lambda }_{1}\\ \cdots \\ \Delta {\lambda }_{n}\end{array} \right]=M\left[ \begin{array}{c}{\Delta v}_{1}\\ \cdots \\ {\Delta v}_{n}\end{array}\right].$$

Further, the variation of target parameters can be solved by the following matrix:

3 $$\left[\begin{array}{c}{\Delta v}_{1}\\ \cdots \\ {\Delta v}_{n}\end{array} \right]={M}^{-1}\left[ \begin{array}{c}\Delta {\lambda }_{1}\\ \cdots \\ \Delta {\lambda }_{n}\end{array}\right].$$

Equation (3) shows that, once the sensitivity and the wavelength change are determined, simultaneous measurements of different parameters can be achieved. However, the multiparameter sensing matrix can only obtain the relative changes of the parameters and cannot determine the absolute values of the parameters. Besides, the prerequisite for using multiparameter sensor matrices is the linear correlation between the measured optical parameters and the resonant wavelength of the spectrum. In the practical environment, there are more of nonlinear relationships between the variables and responses, rendering the application of multiparameter sensing matrices less suitable.

### Machine learning

As revealed by the current research status of optical microcavity, it is worth affirming that optical microcavities have significant advantages in realizing multimode measurements in complex environments with high sensitivity, low detection limit, and high detection accuracy. However, mixing and crosstalk between different resonant modes easily occur, due to the narrow spacing between multiple neighboring modes in the microcavity transmission spectrum. This limits the applications of microcavities in multi-parameter measurements. In addition, manually extracting relevant information about the target to be measured from complex spectra is both time-consuming and subject to human errors.

**Fig. 2:**
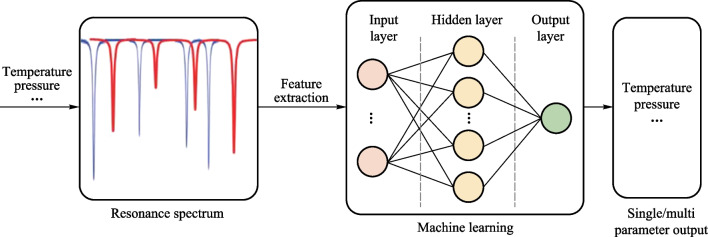
Schematic diagram of sensing principle based on machine learning

The development of a low-complexity, universal multimode detection mechanism is crucial for sensors. Machine learning, as a powerful tool for information fusion and pattern recognition, possesses strong modeling capabilities. Moreover, machine learning is superior to traditional methods in revealing nonlinear dependencies between data. By combining sensors with machine learning, the sensor spectrum can be fully utilized and analyzed. The mapping relationship between sensor information and the target analyte is established. Machine learning-based multi-mode sensing enables the recognition and response to single/multiple parameters, as shown in Fig. 2. Using an optical microcavity as the sensing platform for perceiving single/multiple parameter variations, the multi-mode resonant spectrum changes accordingly with external parameter variations. Then, the multimode resonance spectra collected by an oscilloscope as data samples can be input into the model for training. It is important to note that before model training, data preprocessing is required, such as data denoising and normalization. Also, machine learning algorithms establish mathematical models based on the inputted multi-mode training data. The model consists of three layers: the input layer, hidden layer, and output layer, which are connected by weights [30]. The algorithm can automatically learn the mapping relationship between the input and the output. Typically, this process can be achieved using various machine learning algorithms, including support vector machine algorithms (SVM), decision tree algorithms (DT), random forest algorithms (RF), recurrent neural network algorithms (RNN), and convolutional neural network algorithms (CNN). Finally, the model adjusts its internal parameters through multiple data inputs until it converges to an optimal target. Through these steps, spectral data can be analyzed and processed using machine learning to estimate change parameters from the collective behavior of multimode spectra, enabling single/multiple parameter output. However, it is important to note that machine learning requires a specific quantity of spectral data to train the model to fully explore the mapping relationship between parameter variables and spectra.

## Multimode sensing applications

The basic idea of the optical microcavity sensing mechanism is that small changes in the mode field lead to significant changes in the resonance properties such as resonant wavelength shift and linewidth broadening. At present, research on optical microcavity-based sensors has focused on monitoring changes in individual physical quantities (e.g., temperature, humidity, magnetic field, etc.) [31–34]. Meanwhile, the detection of single molecules or even single ions can be achieved using optical microcavities combined with techniques and mechanisms, such as local plasmon enhancement, laser mode-locking, optical spring effect, and heterodyne detection [35–42]. For example, in 2010, Yang et al. proposed a mode-splitting mechanism that not only achieved the detection of polystyrene pellet particles with a radius of 30 nm but also demonstrated a method for extracting particle size information [37]. In the same year, Xiao et al. theoretically achieved the detection of cylindrical particles based on a mode-splitting mechanism [40]. In 2013, the same group proposed a mode-broadening mechanism to achieve the detection of single nanoparticles and lentiviruses with a radius of 70 nm [43]. In 2016, Baaske and Vollmer used plasmonic gold nanorods modified with a WGM microsphere cavity to detect individual zinc and mercury ions [20]. In 2021, Kohler et al. achieved real-time three-dimensional (3D) position tracking of silica nanosphere particles based on the shifts of three different resonance modes [42].

Although optical microcavity sensors have the characteristics of ultra-high sensitivity and ultra-low detection limit, single-mode tracking limits the detection range so that single-mode detection cannot fully utilize spectral information, resulting in poor sensing accuracy. In addition, the application of a single mode in a complex environment remains challenging, primarily due to multiple effects that often coexist. Traditional microcavity sensing schemes have difficulty in achieving real-time decoupling and independent measurement of multiple parameters. Thus there is an urgent need for multimode optical microcavity sensing technology to address these issues. To this end, researchers have conducted extensive research on high-precision, wide-range multimode single-parameter detection, and multimode multi-parameter parallel detection.

### Multimode single-parameter sensing applications

**Fig. 3:**
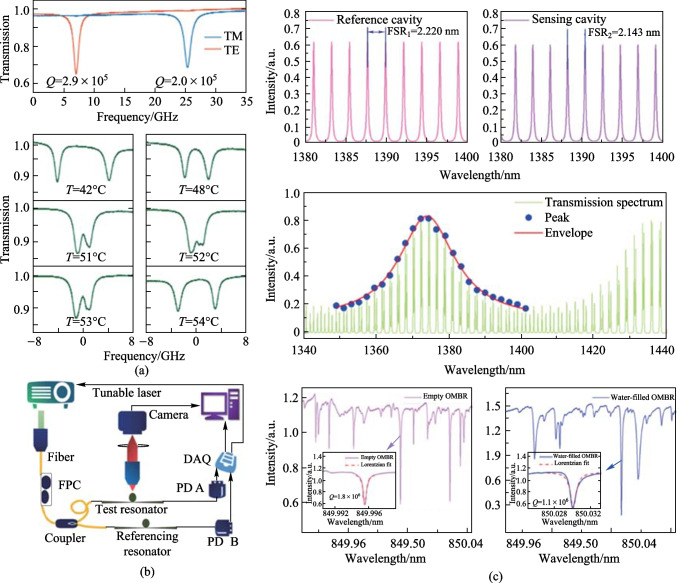
**a** Temperature sensing based on self-reference. Reproduced with permission from Ref. [48]. **b** Biomolecular detection using optofluidic microbubble resonators with an external reference. DAQ, data acquisition card; PD, photodetector; FPC, polarization controller. Reproduced with permission from Ref. [50]. **c** Refractive index measurement based on Vernier effect. Reproduced with permission from Ref. [51].

To address the issue of underutilization of spectral information in single-mode sensing, several multimode single-parameter sensing methods have been proposed. Generally, environmental interference during the sensing process can lead to system instability and can lower the effective detection limit [1, 44, 45]. Therefore, several effective techniques have been proposed to suppress environmental noise and further improve the detection limit. For example, self-referencing sensing methods [46, 47] have been developed. In optical sensors, the resonant frequency of high-quality (*Q*) resonators is typically sensitive to device temperature due to thermal refractive index/thermal expansion effects. Luo et al. proposed self-referenced temperature sensing based on a lithium niobate microdisk cavity, where the self-referencing method selects an additional mode to eliminate the influence of noise [48]. The sensor selects the transverse magnetic (TM) mode and the transverse electric (TE) mode for temperature sensing. When the temperature increases, both modes experience a redshift, but, due to their different temperature responses, the displacement rates of the resonant frequency differ, as shown in Fig. 3a. By mapping the frequency spacing between the two cavity resonance modes to temperature, and by utilizing the frequency shift difference between the two cavity resonances caused by temperature variations, temperature measurement is achieved [49].

However, the detection range of the self-referencing system is constrained by the close spacing (usually a few picometers) between the splitting modes. Thus, Guo et al. proposed an effective bio-molecular detection method using an external reference optical fluidic microbubble resonator, as shown in Fig. 3b. The stability of the tunable laser source as well as environmental disturbances can be monitored by integrating such a resonator into the detection system. The data from the resonator is then used to calibrate the final sensing data, effectively suppressing noise. This method has been used to achieve non-specific detection of bovine serum albumin molecules and specific detection of d-biotin molecules, both at a detection concentration of 1 fg/mL [50]. However, the external referencing method has high preparation requirements, and the sizes and wall thicknesses of the two microbubble resonators need to be as consistent as possible. Currently, the two commonly used optical fluidic cavities are microcapillaries and microbubble resonators. Zhao et al. proposed combining the Vernier effect with an optical microcavity to further improve sensitivity. By integrating a square capillary with a coupled FP microcavity, multiple microfluidic channels can be provided, while also reducing the complexity of the manufacturing process [51]. The Vernier effect is generated by modulation of the spectral envelope due to the mode coupling of two cavities with different free spectral ranges. Refractive index sensing is achieved by monitoring the spectral envelope displacement, as depicted in Fig. 3c.

In practical applications, high precision and large-dynamic-range parameter measurements are also required. Traditional sensing methods rely on tracking the changes of a single mode and can only be achieved by tuning the laser scanning frequency range, with a very limited detection range. Although mode tracking can be continued by fine-tuning the wavelength, this comes at the expense of sensitivity and resolution. Furthermore, traditional sensing methods cannot directly obtain the actual parameter values from the spectrum but instead rely on the relative changes of the resonant modes caused by the parameter variations. Consequently, it is difficult to estimate the absolute value of a parameter solely from the spectrum without knowing its initial value. Recently, multiple resonant modes in WGM microcavities have been used to address the aforementioned issues.

**Fig. 4:**
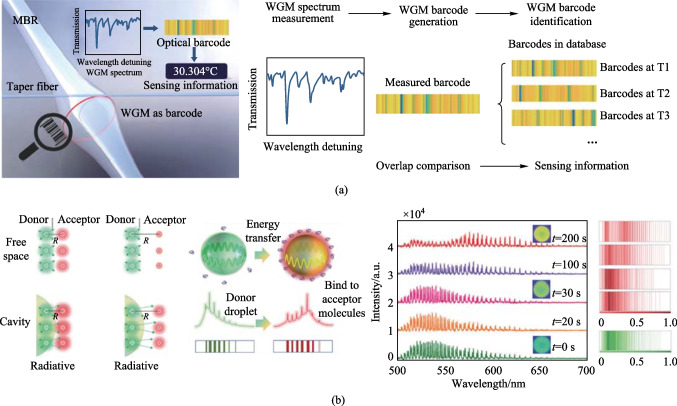
Optical barcodes are used for multimode sensing. **a** Temperature sensing. Reproduced with permission from Ref. [27]. **b** Molecular detection. Reproduced with permission from Ref. [53]

To achieve high precision and large dynamic range detection simultaneously, Liao and Yang proposed an optical WGM barcode technique for temperature sensing, which can monitor the collective behavior of multiple modes and directly read the temperature from the spectrum [27]. The optical barcode relies on the collective behavior of multiple modes in the WGM spectrum, rather than the changes of specific modes. It can provide more information than a single-mode spectrum, such as accurate measurement of temperature. Moreover, due to the randomness of the microcavity, the barcode can be random, or, more rarely, it can be pre-defined to encode some useful information. As shown in Fig. 4a, different temperatures are pre-defined as different barcodes, serving as unique identifiers for temperature recognition. Then, the generated measurement barcode is compared with the pre-defined barcodes, and the similarity is calculated using the cross-correlation function and evaluated using the association function, as follows:

4 $${R}_{xy}\left(m\right)=\left\{\begin{array}{l}\sum_{n=0}^{N-m-1}{x}_{n}+m{y}_{n}^{*}, \quad m\ge 0,\\ {R}_{yx(-m)}^{*}, \qquad \qquad \qquad m<0,\end{array}\right.$$

where *N* is the number of items in the barcode array and *m* is the shift index. If *x*_*n*_ and *y*_*n*_ are similar, then the largest element in *R* is found at the shift value where the elements of *x* and *y* best match. Otherwise, *R* is a null matrix. After determining the best match of predefined barcodes, further refinement of parameter values is done using parameter sensitivity and resonance wavelength shift.

Later, Dong et al. was also based on optical barcode technology, using surface nanoscale axial photonics (SNAP) microcavities for multimode displacement sensing [52]. A barcode was created from the resonance spectrum of the SNAP microcavity based on the distinctive qualities of each order axial mode. To quickly and precisely identify the microcavity displacement, the inter-correlation function technique was used to determine the correlation coefficient between the measured barcode and the predefined barcode. Generally, optical barcodes typically correspond to fixed spectral modes with a single parameter value. Therefore, the ability to monitor the dynamic changes of optical barcodes remains challenging. Zhou et al. proposed molecular detection of dynamic photonic barcodes based on cavity-enhanced energy transfer [53]. As shown in Fig. 4b, the number of receptor molecules that continued to bind to the droplet surface increased as time progressed due to gradient diffusion. As the biomolecule bound to the interface of the photonic cavity, the dynamic spectrum displayed modulated fluorescence spectra shifting from green emission to longer wavelengths. This barcode provided a better method for real-time identification and monitoring of molecular interactions.

Although a pre-defined barcode scheme can achieve high detection accuracy and a wide dynamic measurement range, this method may consume excessive data resources. The detection accuracy highly depends on the amount of spectral data collected, and collecting a large amount of spectral data are often a time-consuming process. Additionally, the WGM-based optical barcode ignores the differences in detection conditions, as the external environment is always prone to fluctuations. Even with small differences, the latest barcode often does not match the data in the pre-defined barcode, leading to significant measurement errors. Moreover, this method does not allow for automatic processing of large amounts of data during measurement. Therefore, an effective method is needed to extract sensing information from multimode resonances.

With the increasing demand for optical measurements, there is a need for more subtle optical features and parameters, and this demand leads to increasingly complex optical data. For example, when using spectroscopy to detect cells, the analysis of biological data poses challenges. Traditional analysis methods are mainly based on the a priori experience of researchers, and so they are time-consuming and prone to human errors. In recent years, artificial intelligence (AI) has made significant progress in various fields. For example, in the field of biomedicine, AI can be applied to medical image processing, disease diagnosis, precision medicine, medical management, and many other areas [54–57]. During this period, the interdisciplinary fusion of photonics and artificial intelligence has also made great progress [58–62]. The outstanding modeling ability and powerful data processing capability of AI can free researchers from tedious and repetitive data processing tasks. Meanwhile, compared to a single sensing mode, the combination of sensors and artificial intelligence effectively integrates multimode sensing information. With a wider dynamic range and lower uncertainty, it provides an important platform for fine measurement [27]. For example, Lu et al. combined an artificial neural network model to achieve voltage detection by extracting multimode transmission depth, with a detection limit 6.7% lower than that achieved by using single-mode detection [58].

**Fig. 5:**
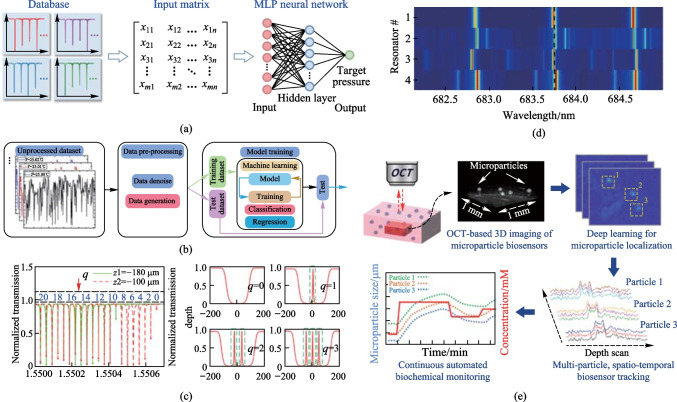
Machine learning for multimode sensing. **a** Pressure sensing. Reproduced with permission from Ref. [28]. **b** Temperature sensing. Reproduced with permission from Ref. [61]. **c** Displacement sensing. Reproduced with permission from Ref. [52]. **d** Refractive index sensing. Reproduced with permission from Ref. [66]. **e** Microparticle-based biochemical sensing. Reproduced with permission from Ref. [67]

As shown in Fig. 5a, Duan et al. constructed a pressure detection system based on microbubble resonators. With the support of a fully connected multi-layer perceptron neural network, they achieved complete spectral feature analysis and improved sensitivity [28]. The results showed that the pressure prediction accuracy reached 99.97% by traversing the spectrum, with an average error as low as 0.32 kPa. Subsequently, Chen et al. achieved highly accurate temperature measurements based on microbubble resonators supported by a generalized regression neural network, with a root-mean-square error of 3.8 × 10^−3^ °C, as shown in Fig. 5b [63]. In addition, Chen et al. theoretically demonstrated that multimode sensing contains more information than single-mode tracking [63]. Dong et al. used a back-propagation neural network to analyze the multi-order axial modes as a function of the coupling position to achieve high-precision detection of displacement [52]. As shown in Fig. 5c, the collective behavior of the multi-axial modes in the transmission spectrum corresponds to the variation of displacement, and this sensing scheme is theoretically feasible. However, the mapping relationship between the multiaxial displacement and the driving depth is complex and highly nonlinear. Dong introduced artificial neural networks to decode spectral data and fit the function relationship between the transmission depth of multi-axis modes and displacement, achieving high-precision displacement measurement. Recently, a reusable biochemical sensor platform in the form of randomly assigned arrays of unmodified glass microspheres has been used to image signals with radiometric WGM in a prismatic excitation scheme [64, 65]. Due to the multidimensionality of the captured signals, interpreting external changes becomes more complex. To address this issue, Saetchnikov et al. successfully achieved refractive index detection using deep learning and fixed-frequency multimode resonator imaging drivers. Within the unit dynamic range (0 to 2 × 10^−3^), the absolute error prediction is on the order of 3 × 10^−6^, demonstrating the prospect of deep learning-based external change quantification, as shown in Fig. 5d [66]. Later, Shah et al. proposed a particle-based biosensor and optical coherence tomography method for remote biochemical monitoring, as shown in Fig. 5e [67]. They modeled stimulus-responsive polymer particles as optical cavities and designed a 3D analysis method to detect submicron changes using optical coherence tomography. As a proof of concept, they demonstrated 3D spatiotemporal tracking of glucose-responsive particles in tissue-mimicking phantoms, which responded to dynamically fluctuating glucose levels. By employing 3D convolutional neural networks, deep learning was further implemented. Automatic processing of continuous 3D time series data streams formed a powerful end-to-end pipeline with great potential for continuous in vivo biochemical monitoring.

### Multimode multiparameter sensing applications

In response to the difficulty of multiparameter sensing by a single mode in a complex environment, multimode spectroscopy methods are adopted to achieve parallel detection of multiple parameters. Currently, multi-parameter sensing based on optical microcavities often relies on sensor arrays [68–71], which consist of several individual sensors for measuring different parameters. In many cases, each optical microcavity is designed to measure specific parameters and requires the surface of the optical microcavity to be modified with sensitive materials. Thereafter, appropriate signal processing methods are used to decouple the different parameters, with the most commonly used information processing method being the multi-parameter sensing matrix.

**Fig. 6:**
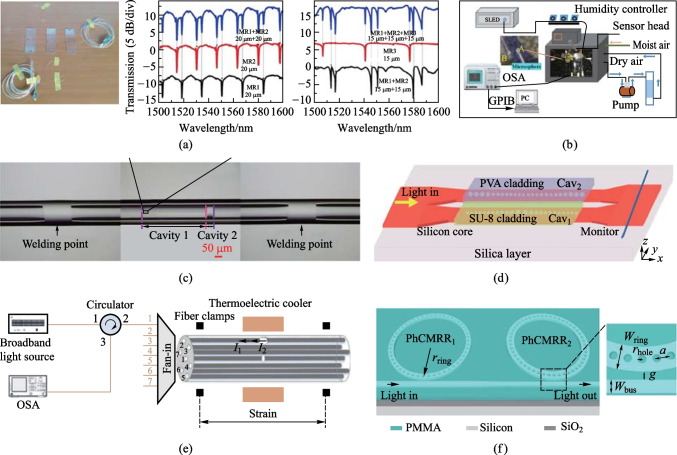
Multiparameter sensing matrices are used for multimode sensing. **a** Stress and temperature sensing based on cascaded micro-cylindrical cavities. Reproduced with permission from Ref. [72]. **b** Ammonia vapor concentration and humidity detection based on cascaded microsphere cavities. Reproduced with permission from Ref. [73]. **c** Compound FP interferometer for measuring high temperatures and pressures simultaneously. Reproduced with permission from Ref. [75]. **d** Temperature and relative humidity can be detected simultaneously using a silicon on-chip with cascading photonic crystal nanobeam cavities. Reproduced with permission from Ref. [76]. **e** Parallel FP interferometers fabricated on multicore fiber for temperature and strain discriminative sensing. Reproduced with permission from Ref. [74]. **f** On-chip simultaneous monitoring of temperature and humidity utilizing error-corrected cascaded photonic crystal microring resonators. Reproduced with permission from Ref. [77]

Kavungal et al. achieved the decoupling of stress and temperature based on a cascaded micro-cylinder cavity sensing platform with a two-parameter sensing matrix [72]. Meanwhile, they cascaded three micro-cylinder cavities on a single fiber cone for the additive validation of the spectra, as shown in Fig. 6a. The results show that the cascaded spectra are roughly equal to the superposition of each spectrum so that each optical microcavity can perform independent parameter measurements. Similarly, Mallik et al. successfully achieved a two-parameter measurement and decoupling of ammonia vapor concentration and humidity in air by using two cascaded microsphere cavities [73]. The device consisted of two WGM microsphere resonators that had been coated with various polymer layers, as shown in Fig. 6b. Due to the exposure of both ammonia and water molecules to the surrounding atmosphere, the optical properties of the coatings changed, resulting in a spectral shift of the WGM resonance. The NH_3_ concentration and relative humidity in the air could be estimated concurrently by monitoring the spectral shift of the related WGMs. However, array sensors cannot independently resolve each parameter without significant cross-talk, which poses limitations and bottlenecks to their application development. Driven by this, there is a great demand for sensors that can accurately detect different parameters. Therefore, Zhang et al. proposed the design of a parallel FP interferometer generated in a seven-core optical fiber that can achieve discriminative measurements of temperature and strain, as shown in Fig. 6e [74]. According to experimental findings, the relative temperature measurement error and relative dye measurement error of the parallel FP interferometer are less than 0.5% and 2.5%, respectively. In addition, Ma et al. designed and experimentally demonstrated a compound FP interferometer for high-pressure and high-temperature sensing based on silica capillaries and optical fibers made from sapphire, as shown in Fig. 6c [75]. The measured gas pressure range was 0 − 4 MPa and the temperature range was 20–700 °C. Ye et al. innovatively proposed a two-parameter sensor that simultaneously detects relative humidity and temperature, and considers the effect of temperature on humidity. Through three-dimensional time-domain finite-difference simulations, they demonstrated the feasibility of simultaneous sensing by focusing on a single output transmission spectrum and using a sensor matrix, as shown in Fig. 6d [76]. The maximum relative humidity and temperature detection errors caused by a 1 pm deviation of the resonant wavelength were only 0.006% RH and 0.026 K. Thereafter, Wang et al. used a cascaded photonic crystal micro-ring resonator to achieve simultaneous humidity and temperature measurements on a chip, as shown in Fig. 6f [77]. The abundance of data in the multiple resonant modes further enhanced the ability of measurement errors to cancel each other out, thus improving the sensing performance reflected by the coefficient of determination(*R*^2^-value), calculated to be 0.97 and 0.99 for RH and temperature sensing results, respectively.

**Fig. 7:**
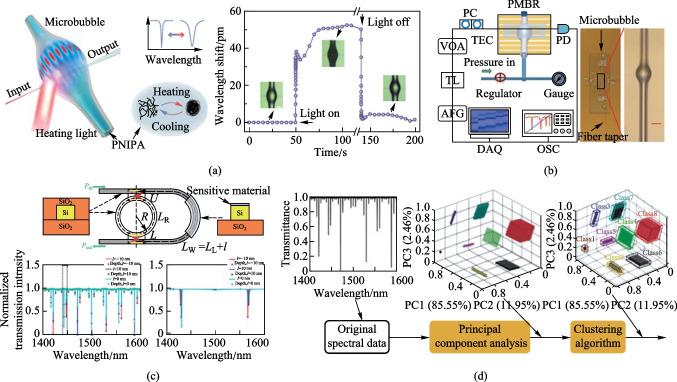
**a** Simultaneous detection of temperature and refractive index. Reproduced with permission from Ref. [26]. **b** Temperature and pressure detection based on a single microbubble cavity. Reproduced with permission from Ref. [25]. **c** Machine learning-based multi-parameter sensing in a multimode self-interference micro-ring resonator. Reproduced with permission from Ref. [86]. **d** Unsupervised gas classification by multimode micro-resonator. Reproduced with permission from Ref. [87]

Although sensing arrays have achieved multiparameter sensing using wavelength division multiplexing, their complex array structure and detection cost hinder their further development in the case of larger numbers of target parameters. Thus, Duan et al. achieved dual-parameter measurements of temperature and refractive index by using a single microbubble resonator sensing platform with a self-referencing sensing mechanism, as shown in Fig. 7a [26]. They monitored the leap kinetics of a typical phase change material poly-n-isopropylacrylamide (PNIPA) using high-*Q* optical flow microcavity experiments. The integrated microfluidic channels provided effective coupling between the PNIPA molecules and the resonant optical field for operando detection. Usually, modes of different orders have different field distributions. Duan et al. picked a radial third-order mode as the sensing mode and the fundamental mode as the reference mode, and the two modes showed different responses during the phase transition. They used a dual-mode self-reference technique to extract the temperature and refractive index changes of PNIPA from the microcavity resonance spectra during the phase transition. This gave them a fantastic opportunity for on-demand investigation of dynamic biological processes. However, the above work required a rigorous selection of modes for multiparameter sensing. Later, Wu et al. in the same group also used multiple modes of a single microbubble resonator to achieve independent measurement and real-time decoupling of temperature and pressure, as shown in Fig. 7b [25]. This work did not require a strict mode selection, but only a certain interval between modes, which could be more easily selected for two modes and combined with a two-parameter sensing matrix to achieve two-parameter sensing. Optical microcavities provided powerful tools for the development of fast and accurate physical quantity sensing techniques. The inherent inertness of such primitive microresonators, however, prevented their widespread use in new applications, including gas detection. In this case, chemical functionalization can enhance the capabilities of sensing applications [78]. Therefore, Yao’s group implemented two-dimensional-material functionalized microcomb sensors by asymmetrically depositing graphene in over-modal microspheres [79]. Spectral capture of Stokes solitons belonging to different transverse mode families could be co-produced in a single device using a single pump. These Stokes solitons with locked repetition rates but distinct offsets could create ultrasensitive taps in the electric domain and have unique benefits for selectivity and individual gas molecule detection. Finally, they achieved detection of three gases by a third-order sensing matrix. Later, the group deposited graphene on erbium-doped over-modal microspheres to realize functionalized microlaser sensors [80]. Multiple laser lines are excited in various mode families of a single micro-resonator using 980 nm pumping. Interference between these splitting mode lasers due to graphene-induced intracavity backscattering produces beat notes in the electrical domain (0.2–1.1 MHz) with sub-kHz accuracy. Finally, the identification of the four gases is achieved by a fourth-order sensing matrix.

Most of the above work is based on the sensing method of the multiparameter sensing matrix, which is no longer applicable when there is a nonlinear relationship between the sensing parameters and the sensing modes. Therefore, a more general data processing method is needed. The powerful nonlinear modeling capability of machine learning allows for multidimensional data processing and analysis of spectra [81–85]. Recently, Hu et al. proposed a self-interfering micro-ring resonator multimode sensing method that uses artificial neural networks to process the signal for the identification of two gases, as shown in Fig. 7c [86]. The self-interfering micro-ring resonator (SIMRR) allows multimode sensing over a wide wavelength range but is not affected by frequency noise. For parameter estimation, transmission depths of various resonant modes can be gathered using a tunable probe laser excitation detection device. The training and test phases of a back propagation neural network are utilized for signal processing. The training and test data are created from these transmission depths over a range of wavelengths. Two gas sensors were numerically validated using SIMRR multimode sensing as an example. However, backpropagation neural networks, forming a supervised learning algorithm, require large training data sets, and labeled data are often difficult to obtain in practical applications. Thus, unsupervised algorithms are a form of learning used without any training data or guidance, eliminating the need for creating large amounts of labeled data. It is also capable of discovering new patterns in the training data set, some of which can even go beyond prior knowledge and scientific intuition. Thus, Zhang et al. developed another high-precision unsupervised classification model in multimode SIMRR to achieve the identification of three gases, as shown in Fig. 7d [87]. The developed sensor was used to numerically validate the unsupervised classification algorithm. The numerical findings demonstrate that for the specified three-gas sensor with a signal-to-noise ratio larger than 60 dB, the classification model has extremely high classification accuracy.

## Conclusion and outlook

This paper presents the basic characteristics and sensing mechanisms of multimode sensing based on optical microcavities, briefly introducing multimode sensing methods such as multiparameter matrices and machine learning. The paper also presents the applications of multimode microcavity sensing in different fields. The current research on multimode single-parameter focuses on improving detection limits and achieving wide-range sensing measurements, such as in noise suppression using self-referencing techniques, and in application of optical barcodes for achieving high accuracy and wide-range sensing measurements. The research on multimode multi-parameter sensing focuses on techniques, such as multi-parameter parallel detection and independent decoupling based on sensor matrix, and intelligent multi-parameter sensing based on machine learning.

In recent years, intelligent optical multimode sensors have made significant progress due to interdisciplinary collaboration, aiding researchers in overcoming the complex data bottleneck in multimode sensors. By leveraging machine learning algorithms, the fusion of multimode sensing information can be effectively achieved, further enhancing sensing resolution and sensitivity. Currently, it is widely recognized that ensuring accurate prediction models in machine learning algorithms requires a large amount of high-quality data. However, due to the limitations imposed by many real-world conditions, it is often challenging to obtain sufficient data. One effective approach is to use machine learning algorithms to generate synthetic data using techniques like generative adversarial networks (GANs) to supplement training data. In addition, in future work, it is expected that a general-purpose sensing model can be realized by optimizing the model. When the sensing model is applied to different sensing platforms, there is no need to retrain the model, which lays the foundation for the development of multimode intelligent sensing.

Meanwhile, there are still other sensing methods being studied in multimode sensing. For example, the recently widely discussed optical frequency comb consists of a series of equidistant and highly stable frequency lines. As the basis of the most accurate frequency standard in the world, optical frequency combs have been widely used in precision measurement fields, such as fundamental physical constant measurement, optical atomic clocks, and molecular spectroscopy. Undoubtedly, optical frequency combs provide new opportunities for single/multi-parameter measurement. In the future, we can expect the integration of sensors with different sensing methods, to achieve more reliable multimode sensing for sensing applications in complex environments.

## Data Availability

Data underlying the results presented in this paper are not publicly available at this time but may be obtained from the authors upon reasonable request.
